# Virtual reality for visions (VRV): a proof-of-concept study examining the development of a new treatment for distressing visual hallucinations in people with psychosis

**DOI:** 10.1136/bmjopen-2025-107535

**Published:** 2026-01-12

**Authors:** Robert Dudley, Samuel Sargeant, Christopher Gibbs, Louise Prentice, Laura McCartney, Charlotte Aynsworth, Morag Maskey, Andrew Skeggs

**Affiliations:** 1 University of York, York, UK; 2 Cumbria Northumberland Tyne and Wear NHS Foundation Trust, Newcastle upon Tyne, England, UK; 3 The Open University, Milton Keynes, UK; 4 Northern Design Centre, XR therapeutics, Gateshead, UK; 5 Royal Derby Hospital, Derby, UK

**Keywords:** Schizophrenia & psychotic disorders, Virtual Reality, Feasibility Studies

## Abstract

**Introduction:**

Visual Hallucinations (VHs) (seeing things that others do not, or visions) are a common feature of psychosis, causing significant distress and disability. Services rarely ask about these important experiences, and crucially there are no proven beneficial psychological treatments. There are at least two key challenges faced when treating VHs. First, people report not knowing why they see things others don’t, which leads them to feel alone and different from others. Second, they feel they cannot trust their own eyes to tell what is real or not, which can lead to fears they will be hurt or harmed by the VH, or even if they know the experience is not real, they may fear that they are losing their mind, or that they are not able to control or manage their experiences. For these reasons, they may struggle to put skills and strategies into practice when in the presence of the VH. Consequently, we have developed a novel treatment that addresses these core issues. First, we have a psycho-education and coping strategies package called Visual Unusual Sensory Experiences (VUSE) that uses the best aspects of digital technology (animations, videos) to explain why people have VHs and provides normalising information to help the person to feel less alone. It introduces coping strategies that are then tested in Virtual Reality sessions (VR for Visions VRV) where a representation of the visual experience is provided, enabling the person to safely develop skills and gain a sense of mastery and empowerment. We now plan to test this approach in a proof-of-concept study to help determine if this will help people use these skills in the real world and so help reduce distress, improve functioning and quality of life. We will address uncertainties in the feasibility of developing and delivering this treatment and inform its future use in a larger trial.

**Methods and analysis:**

The study is a single arm feasibility trial (n=16) evaluating VUSE+VRV and treatment as usual. The study is recruiting people with psychosis and distressing VHs in one NHS Trust and uses independent but non-blind research assistants to undertake assessments before, during and after treatment (at baseline, 6, 12 week) and at follow-up (16 weeks). Quantitative information on recruitment rates, adherence and completion of outcome assessments (VHs, other psychiatric symptoms, quality of life and perceived recovery) will be collected. Qualitative interviews will capture service-users’ experience of therapy. Analyses will focus on feasibility outcomes and provide initial estimates of intervention effects. Thematic analysis of the qualitative interviews will assess the acceptability of the intervention.

**Ethics and dissemination:**

The trial has received NHS Ethical and Health Research Authority approval (25/EM/0077). Informed consent will be obtained from all participants. Findings will be disseminated directly to participants, and services as well as through open access peer-reviewed publication(s).

**Trial registration number:**

ISRCTN11350954.

**STRENGTHS AND LIMITATIONS OF THIS STUDY**

- The virtual reality for visions (VRV) trial is a new treatment for distressing visual hallucinations.
- The treatment targets causal factors implicated in hallucinations.
- Patient Public involvement has shaped the development of the treatment, design and running of the study.
- There is no control group, meaning we do not know what factors contribute to any change.
- The follow-up period is short, meaning that we cannot determine whether any benefits endure.

## Introduction

 Visual hallucinations (VHs) are the experience of seeing things that are not actually there.^1^ They are common in people with psychosis, with around one third of people reporting VH.^2^ Interviews with people with VH and psychosis have revealed how distressing their experiences are: *“I want to disappear from the earth; if I disappear, then maybe I won*’*t see the visions. If I could just spend my whole life in a coma, I would pick that any day of the week rather than live the life that I have right now”.*^3^ This experience is not unusual, as VH are some of the most distressing and disabling symptoms of psychosis^4^ and are associated with reduced quality of life, and more frequent and prolonged hospital admissions.^5^

There is a clear need for help, but existing treatments including medications and treatments like Cognitive Behavioural Therapy for Psychosis (CBTp) don’t seemingly benefit visions.^1^ To date, only a limited number of studies have specifically addressed treatment of VH in psychosis. Case reports have used medications,^6^ transcranial stimulation^7 8^ or talking treatments.^9 10^ Two case series have used CBTp and found meaningful reduction in idiosyncratic measures for VH, but the lack of validated outcome measures for VH^11^ means it is uncertain if there were demonstrable benefits.^12 13^ A recent study used imagery focused treatment with promising results, but was also limited by being a single case series^14^ and the lack of sensitive, validated outcome measures.^11^

To try and improve treatment, we have drawn on the updated guidance from the Medical Research Council (MRC) Complex Intervention Framework.^15^ Phase one in the development of an intervention aims to describe how an intervention is expected to lead to its effects and under what conditions, and the key mechanisms of the intervention.^16^ Our work draws on research that has explored the phenomenology of VH,^3 17 18^ how common they are^19^,^22^ and what leads to people seeing things others do not, clearly identifying the role of mechanisms such as sleep^23^ trauma, dissociation^17 24^ and mental imagery.^25^ We have also developed valid interviewer^26^ and self-report outcome^27^ measures to aid the appropriate evaluation of interventions.

Through these efforts, we have identified two core issues that make VH so very impactful. First, people struggle to understand why they see things that others do not, and so are fearful as to what it means that they have these experiences. From the person’s perspective, it makes them feel different from others as they believe others do not have these experiences. Second, at the heart of the experience of VH is the inability to tell if what they are seeing is real or not. This is deeply troubling, as you seemingly cannot trust your own eyes. This leaves people believing that if it is real, then there could be a threat to their physical well-being (they worry they or those they care about could be hurt by the VH). If they believe it is not real, then this is a threat to their psychological well-being as they would be fearful they are losing their mind for seeing things others do not.^28^ Finally, even if they recognise the experience as a VH and do not fear for their sanity, they may lack effective strategies to manage or control the experience and so are concerned their future will be blighted by ongoing VH. These appraisals lead the person to feel unsafe and different to other people, leading to distress, stigma, isolation and a reduced quality of life. To address these core issues, we need a treatment which helps people understand what can cause VH, that other people have similar experiences and that they do not need to be afraid. The person also needs to learn to manage their VH in the presence of these experiences. To do this, we need a method to bring VH into the therapy room and to help people build confidence and skills in dealing with the VH differently; to help them to safely test their fears before applying this to real-world settings. This should enable people to trust their own eyes, help them distinguish what is real from what is not, and to feel safer.^29^

Our treatment directly addresses these issues by increasing understanding using specific psychoeducation about VH and actively practising coping strategies using virtual reality for visions (VRVs). Our psychoeducation package explains why people see things others do not and provides a normalising account of these experiences with illustrations of how common these experiences are, to help the person feel less alone or different from others. It provides testimonies of people who have learnt to live well even when their VH have not gone away, helping to promote hope. It also illustrates how a number of known factors (isolation, sleep, dissociation) can increase or decrease the chance of seeing things others do not. This helps people realise that their VH happen for understandable reasons which may in turn help give them greater control over their own experiences. We have developed this package as part of the Managing Unusual Sensory Experiences (MUSEs) treatment.^30^ One module of MUSE is specifically focused on VH, titled Visual Unusual Sensory Experiences (VUSE). It is brief (4-6 one hour weekly sessions), uses engaging animations and video clips, and runs on NHS laptops without the need for WIFI, meaning it is easily accessible. A number of studies, including randomised controlled feasibility studies of MUSE in people with first episode psychosis^31^ and people with At Risk Mental State (a group with a higher risk of transition to psychosis^32^), showed good uptake, adherence, high satisfaction ratings, promising therapeutic effects and no adverse reactions.

The second key component of our approach is to use VRV to provide a safe environment to actively test, challenge and change the way people deal with VH. Virtual Reality is a simulated 3-dimensional environment with scenes and objects that people can explore and interact with. This can create an immersive experience that triggers emotional responses similar to those in real-world situations but in the safety of a clinic. Systematic reviews^33^ and recent work with people with paranoia in the context of psychosis^34^ have found that VR is popular, accessible and helpful.^35 36^ VR provides the perfect resource to working with VH as VR allows the person to use the ideas learnt in VUSE to be repeatedly and safely practised in the presence of a representation of the feared vision, enabling confidence to be built before trying these in the real world.

### Research question

This study asks if it is feasible to develop and deliver a novel digital treatment combining high impact, VH specific, psycho-education (VUSE) with the powerful and engaging experience of Virtual Reality (VRV) for VH in people with Psychosis. It addresses the feasibility of delivery and uncertainties about the intervention itself by using a case series to explore issues around optimal content, delivery, acceptability and adherence of VUSE+VRV to inform a future feasibility trial.

## Methods and analysis

### Trial design

The design uses a case series as a proof-of-concept study. Participants (n=16) will have 4–6 sessions of psycho-education (VUSE) and then 4–6 sessions of VRV provided by trained and supervised psychological therapists.

Assessments will be conducted at baseline, mid (post VUSE at 6 weeks) and post treatment (after VRV at 12 weeks) and at follow-up (16 weeks with an allowance of another 4 weeks if needed) by independent but not blinded research assistants. This will determine if the intervention is acceptable, engaging and whether it reduces distress and improves quality of life. Qualitative interviews will be used with participants to inform the future development and use of the intervention (detailed in Protocol v1 13/01/2025).

### Randomisation and blinding

There is no randomisation or blinding as this is a case series. The researcher undertaking assessments will be aware of allocation but is not delivering the treatment and will not review notes or discuss treatment with the therapists during the intervention stage.

### Participants

The trial participants will be people with psychosis and self-reported difficulties related to seeing things others do not (VH). We will recruit 16 people (aged 16 and over) from Community Mental Health Treatment Teams (CTTs) and Early Intervention in Psychosis (EIP) services in Cumbria, Northumberland, Tyne and Wear NHS Trust (CNTW).

Following referral by the clinical team, all people interested in taking part will then be approached by the research team, given information about the trial (see online supplemental materials 1), and then screened and assessed for inclusion in the trial. All participants will be given at least 24 hours to consider participation. Written informed consent will be obtained from each participant prior to any participation.

The inclusion criteria are: have a history of VH for at least 3 months; would like to receive a psychological intervention specifically for VH; aged 16 and over, receiving care from EIP or CTT; meets ICD11 criteria for schizophrenia, schizoaffective disorder or diagnosis of schizophrenia spectrum psychosis (F20-29) or entry criteria for an EIP service; have the capacity to provide written informed consent; be judged by their clinician to be clinically stable for the preceding 4 weeks.

The exclusion criteria are: any intellectual disability or severe cognitive dysfunction precluding their ability to provide informed consent; any primary diagnosis of substance misuse dependency or traumatic brain injuries, organic psychoses or dementia; unable to use VR owing to epilepsy, poor eyesight or dizziness.

### Assessments

Basic demographic and clinical data will be collected (eg, age, gender, ethnicity, clinical diagnosis) at baseline. Clinical outcomes including VH, psychosis and affective symptoms, functioning and quality of life will be assessed at all four time points (baseline, mid treatment (post VUSE sessions at 6 weeks), post VRV intervention (12 weeks) and follow-up (16 weeks).

In terms of primary outcomes, the presence of distressing VH (and their frequency, duration, content) will be confirmed using the North East Visual Hallucination Inventory (NEVHI^37^). The distress and impact of the VH will be measured using the Psychotic Symptoms Rating Scale^38^ adapted for use with visions^26^ and a self-report measure the Hamilton Programme for Schizophrenia Voices Questionnaire^39^ which has been adapted for use with people with VH.^14 27^ In terms of secondary outcomes, hallucinations in other modalities will be collected for lifetime occurrence using the Multi-Modality Unusual Sensory Experiences Questionnaire^40^ and for the past month using the Multimodal Hallucinations Measure.^17 21^

Other secondary outcomes include quality of life assessed using the Questionnaire about the Process of Recovery,^41^ functioning (DIALOG^42^), paranoia (Green Paranoid Thoughts Scale revised^43^), levels of depression (Patient Health Questionnaire-9 PHQ9^44^) and anxiety (Generalised Anxiety Disorder Scale^45^) and stigma (Internalised Stigma of Mental Illness Scale^46^). Service use data will be collected with an adapted version of the Client Service Receipt Interview^47^ and experience of therapy assessed using the working alliance inventory^48^ and satisfaction with therapy (Satisfaction with Therapy and Therapist Scale-Revised^49^) will be undertaken at the end of the treatment period. Data will be reported in line with the Consolidated Standards of Reporting Trials (CONSORT)—Social and Psychological Interventions (CONSORT-SPI) statement^50^ and Standard Protocol Items: Recommendations for Interventional Trials (SPIRIT) guidelines.^51^ This guidance recommends minimising the distinction between primary and secondary outcomes so all outcomes will be reported at the end of the trial.

### Data management

All documents, including electronic files, will be stored confidentially. All trial data will be entered on paper and transcribed or entered directly to a secure purpose-made Excel spreadsheet. This spreadsheet will be independently checked for accuracy against the validated questionnaires. The trial investigators, trial oversight committee and sponsor will have access to the full data set.

### Analysis

As this is a proof-of-concept study, there is no distinction between primary and secondary outcomes as these measures are to help identify important parameters for a future trial (ie, completion rates and selection of best outcome measures see CONSORT—Social and Psychological Interventions (CONSORT-SPI^52^) statement, and SPIRIT guidelines.^53^ All outcomes will be reported at the end of the trial.

All statistical analysis will be primarily descriptive and will generate means and SD and/or medians and IQRs for the feasibility outcomes of interest. Initial efficacy will be evaluated through repeated measures ANOVAs to provide change scores between baseline, mid, post-therapy and follow-up. The focus is on effect size/CI estimations; therefore, no p values will be reported. The analysis plan will be developed for the outcome measures and agreed with the oversight committee before the end of data collection and a copy uploaded to the trial’s registry in advance of any analysis. Therapy session by session ratings (frequency, distress, impact of VH) will also be plotted to examine in more detail treatment effects for VH.

### Sample size

As the main objective is to establish feasibility parameters for a definitive trial, there are no formal sample size calculations, interim analyses or stopping rules.

### Qualitative research

Qualitative interviews will be used with approximately 12 participants to inform the future development and use of the intervention. The interviews will consider whether the VUSE and VRV treatment was acceptable, engaging and relevant. A particular focus will be on the acceptability and experience of the virtual reality. Participants will be invited to be involved in the interviews after the end of treatment at the 12-week assessment point, and interviews will ideally be conducted before the 16-week scheduled assessment but can be undertaken up to 20 weeks from enrolment in the study if there are issues in arranging visits etc. Topic guides for semi-structured interviews will be co-developed with the Patient Public Involvement (PPI) group and piloted in advance. Participant numbers should provide sufficient data to meet criteria for trustworthiness of the findings.

### Qualitative analysis

Qualitative data, gathered from the structured interviews with participants, will be analysed using an inductive thematic analysis.^54^ This form of analysis allows for both basic and more complex themes to be classified from interview data (based on categorising sections of transcripts), based on the data themselves rather than a prior theory. Transcripts will be coded by the research assistant and qualitative lead. Quotes to illustrate each category will be selected. Understanding of the data will be developed with PPI members.

### Criteria for proceeding to a full trial

We will use criteria for assessing study success and identifying feasibility factors required for delivering the definitive study and follow a systematic process for decision making after pilot and feasibility trials which helps identify criteria used to go to a full trial.^55^ These criteria are being developed with the PPI group to help determine if a full trial is warranted but will likely include participant recruitment, adherence with the intervention, retention of participants, intervention fidelity and acceptability to assess the trial. This will use both quantitative and qualitative data derived from the study. The progression criteria will be divided into three categories (green, red and amber). Areas that are amenable to change before a feasibility trial will be investigated and solutions discussed with the PPI group for acceptability. This will help consider if a further trial is timely, necessary and deliverable.

### The VUSE and VRV intervention

The treatment will be delivered by a qualified, trained and supervised psychological therapist. The person with VH will be seen face-to-face. The treatment consists of three stages described below.

#### Description of the VH

The initial session elicits a detailed description of the VH which will be shared with the VR programmers to allow a person-specific representation of their VH to be developed. The person identifies a visual experience they want to work on in the VR, one that causes distress and impact and is seen frequently enough to be helpful to work on. People with visions typically report seeing faces, people, figures, animals and objects (^19 20^which are also captured on the NEVHI^37^). The assessment is used to create a story board that first describes the setting in which the vision is seen (a living room, bedroom or garden, for example). Key information about the layout, background noises, lighting, time of day etc, is captured. Next, information about the vision (size, shape, colour, movement, sounds, facial features etc) is asked about and the person is encouraged to help represent this by drawing or describing the image, or using images from the internet. Finally, any additional information that makes the vision more impactful is captured, such as it moving towards the person or looking at them in a particular way (see online supplemental materials 2).

#### VUSE

During the programming time (~3 weeks) the therapist and service user work through the VUSE package over 4–6 weekly sessions (~60 min per session), to understand the causes of VH and foster new skills for their management. The treatment draws on the most helpful and valued modules from the MUSE package and has been developed further by PPI input to cover:

#### What are visions?

This provides normalising information about the frequency of VH (or the term used in the package is visions) and the factors that tend to increase visions (for example, isolation and sleep deprivation), along with testimonies from other people with visions providing powerful messages that the person is not alone, and that recovery from or living well with visions is possible.

#### How the mind works

This module explains what can lead to people seeing things others do not. It explains how our visual perceptual system can lead to mistaken perceptions, and it can lead to predictable and expected errors or illusions.^56^ For example, how we easily see faces in everyday objects as a result of our brains being equipped with a neural circuitry specialised for preferentially processing faces.^57^ This places visions in the context of being normal in that they result from a perceptual system that can be put under strain by processes like poor sleep or higher stress, which can increase the risk of making these sorts of mistakes. Key psychological processes such as heightened visual imagery, trauma, expectancy and threat detection are explained with engaging videos and animations. For example, the person may be provided with information about how memories from traumatic events are more likely to be experienced as intrusive memories without contextual cues and can therefore be experienced as belonging to the here and now. Based on these understandings, targeted coping strategies are introduced.

#### Assessment and formulation: identifying triggers, unhelpful appraisals and coping

This module starts by considering whether there are patterns or identifiable triggers/contexts associated with seeing visions. Commonly, they are experienced when tired or stressed, often when the person is alone or in the dark. Identifying these triggers can lead to simple changes (like substituting different activities or turning on lights at the time the vision is experienced) that can reduce the frequency or impact of visions. Next, key appraisals related to seeing the vision are elicited. These may be thoughts of harm to self or others as the vision represents a real threat. If the person perceives the vision as not real, then they may have fears of losing one’s mind. Other appraisals are that the visions will persist, or it may be that the content of what people see is distressing. These appraisals and resulting behaviours (leaving the room where they see a vision, trying to look away from the vision) are then linked to more helpful and constructive behaviours that are identified and practised in preparation for the VR session.

#### VRV

Following the VUSE sessions, the person attends the VR suite, which is a comfortable environment with a large screen on which the VR VH is displayed. The preference is to use the VR suite, but the option of using a VR headset (at their home or team clinical base) is possible and will be discussed with the participant. The person will have 4–6 sessions with the therapist present. There are four potential treatment approaches feasible within the VR environment. The first is exposure to the representation of the vision. This may help the person habituate to the presence of the VH and overcome the common tendency to leave when in the presence of the VH. Second, the participant will be encouraged to try behavioural experiments where they are supported in testing if the vision is real, perhaps by paying more attention to it and noticing characteristics that challenge if it is a real phenomenon (such as it having unusual features, or clothing). They will be further encouraged to test if it is real by practising throwing an object at it, or trying to photograph it on their phone. Third, people will be encouraged to explore safety beliefs, so that they practise dropping safety behaviours and defences, perhaps by turning their back on the vision to help show they are safer than they realise. Fourth, using the power of the VR environment, we can help the person develop control over their experiences by practising imagery transformation techniques that are commonly used in working with post-traumatic stress disorder or social anxiety to help change the power and intensity of the visual experience (such as changing its size or colour). The sessions will be recorded using video recording equipment in the VR suite (this will not be done if the person is using the headset option or is away from the VR suite). The therapist and participant will review these together after the session to enable the person to consider what they have learnt or done differently in the VR session, and a copy of the video is available to the person to take home and review to help them better manage when they see the vision away from the VR suite.

The therapist will call the participant in between scheduled sessions to check on progress with homework and to check on well-being.

### VR equipment

The XR/CBT Immersive Studio is a Class I Software as a Medical Device specifically developed to support healthcare professionals in conducting CBT and/or other talking therapies to support treatment of anxiety, phobias, depression and other mental health disorders. Through immersive scenes replicating anxiety-inducing scenarios, it creates controlled environments for patients to address their anxieties. The primary aim is to expedite patient recovery, particularly where graded exposure is a part of treatment.

### Details of the VR software

The VRV software application is composed of a set of virtual environments, including different scenes created using 3D models, ambient audio and 3D computer characters, with animations and speech. The environments are driven by source code which handles the logic of the programme, the behaviour of the computer characters, as well as the user interaction and data storage. The code is implemented on top of XRT background libraries and third-party libraries. The software is built using Unity (Unity Technologies). Unity acts as a render engine, displaying the virtual environments to the user through the VR suite or the headset.

### Treatment as usual

Participants will continue to receive their usual care which will typically consist of long-term prescription of psychiatric medications and meetings with a mental health practitioner. No restrictions will be placed on TAU though involvement in other psychological therapy will be discussed with the person and the team in case it leads to challenges in attending frequent appointments. We will collect detailed data on treatment as usual.

### Oversight

As the trial is unblinded and not randomised, a single trial oversight committee will combine the functions of a Trial Steering Committee and Data Monitoring Committee. The committee consists of an independent Chair, clinician, statistician and person with lived experience. There is a separate PPI group facilitated by two co-applicants for the study with lived experience of psychosis. The trial will be audited by the Sponsor at first participant and annually.

### Safety

In order to monitor for adverse events (AEs), we maintain close links with the participant’s clinical team throughout, have a staff member present throughout while the VR equipment is in use, and record events that we become aware of during a participant’s participation. We will also check the participant medical notes prior to each study visit for the following events pre-specified as adverse: (1) All deaths, (2) Suicide attempts, (3) Serious violent incidents, (4) Admissions to psychiatric hospital/secure units and (5) Formal complaints about therapy.

#### Definitions of adverse events

AEs are any untoward medical occurrence, unintended disease or injury, or untoward clinical signs in participants, whether or not related to the intervention or assessments. This includes AEs related to the VR intervention and also to all research procedures involved. We note that a temporary increase in anxiety symptoms is expected in any psychological treatment involving confronting a feared situation and this would not be considered an AE. All reportable AEs will be reported from the baseline visit until completion of the 16-week follow-up point.

#### Definitions of serious adverse events

A serious AE is defined by the ISO14155:2011 guidelines for medical device trials as an untoward medical occurrence that: results in death; is a life-threatening illness or injury; requires (voluntary or involuntary) hospitalisation or prolongation of existing hospitalisation; results in persistent or significant disability or incapacity; medical or surgical intervention required to prevent any of the above; leads to fetal distress, fetal death or consists of a congenital anomaly or birth defect or is otherwise considered medically significant by the investigator.

Life-threatening in the definition of a serious AE (SAE) refers to an event in which the subject was at risk of death at the time of the event. A planned hospitalisation for a pre-existing condition, without a serious deterioration in health, is not considered to be a serious AE. Admissions to psychiatric hospitals are not unexpected in this client group.

#### Causality

The relationship between the intervention (VUSE, VRV) or other research procedure (ie, assessments and generation of the VR representation) and the occurrence of each AE will be assessed and categorised. The chief investigator will consider a range of potential causes (such as the history and nature of the participant’s underlying condition, other treatments/interventions/therapy, current life events and risk factors etc) when trying to determine any causal relationship. The investigator will make an initial assessment of whether the SAE is potentially related to the intervention or trial procedures, and the expectedness, and report as necessary to the regulatory authorities within the appropriate timescales (eg, related and unexpected SAEs) to the Sponsor, research ethics committee and adverse incidents to the Medicines and Healthcare products Regulatory Agency. The oversight chair (an independent clinician) will review decisions about relatedness and expectedness, and they will be reported at the next oversight meeting.

### Patient and public involvement

Patient and Public Involvement is facilitated by two co-applicants with personal experience of psychosis. The current study protocol and MUSE/VUSE intervention were developed in collaboration with people (and their carers) who have lived with hallucinations. A fully funded PPI group of 6 members has been formed in line with recommendations of advisory groups that encourage service-user involvement in NHS research (http://www.invo.org.uk/). The PPI group is actively involved in all stages of the research programme, for example, advising on participant recruitment, information sheets, staff training, treatment development, topic guides, analysis of the qualitative interview data and dissemination of the research findings. Meetings are held online and/or in person approximately monthly. An independent PPI representative also sits on the oversight committee.

## Ethics and dissemination

The trial received ethical approval from the NHS East Midlands-Leicester South Research Ethics Committee (25/EM/0077) Health Research Authority (HRA/HCRW) approval (IRAS 330558). CNTW NHS Foundation Trust is the trial sponsor. All participants will give informed consent. Recruitment is due to start in July 2025 for approximately 8 months. Any changes to protocol will have sponsor and ethics approvals. The study is due to complete in June 2026, and the results of the trial will be published in a peer-reviewed journal and made open access.

## Supplementary material

10.1136/bmjopen-2025-107535 online supplemental file 1

10.1136/bmjopen-2025-107535 online supplemental file 2
