# Supplementary material for: Virtual reality for visions (VRV): a proof-of-concept study examining the development of a new treatment for distressing visual hallucinations in people with psychosis
Source: BMJ Open. 2026 Jan 12;16(1):e107535. doi: 10.1136/bmjopen-2025-107535 (PMC12815111; doi:10.1136/bmjopen-2025-107535)
Supplement: online supplemental file 1 [file bmjopen-16-1-s001.docx]

Appendices

Patient Information sheet for trial

**PARTICIPANT INFORMATION SHEET FOR VIRTUAL REALITY FOR VISIONS (VRV):**

| **Version: 3.0** | **Date: 19/05/2025** |
| --- | --- |

**Study Title:** Virtual Reality for Visions (VRV): a proof-of-concept study examining the development of a new treatment for distressing visual hallucinations in people with psychosis

**Chief Investigator:** Dr Robert Dudley

We would like to invite you to take part in a new research study called Virtual Reality for Visions (VRV). Before you decide, we would like you to understand why the research is being done and what it would involve for you. Please read this information sheet and discuss it with others, if you wish. One of our team will contact you in a few days and go through the information sheet with you and answer any questions you have. Ask us if there is anything that is not clear, or if you need more information. This is your copy of this information sheet, for you to keep for future reference.

**Why have I been invited?**

You are being invited to take part because you have let your care team know you have unusual sensory experiences like seeing things that others do not, because you have these experiences at least once per week, for three months or more and because you are aged 16 years or over.

**What is the purpose of the study?**

We are interested in seeing how well a new therapy for distressing visual hallucinations (sometimes called visions) works. We have developed a treatment that tries to help people understand and manage these experiences.

This study is part of a new project on the use of immersive virtual reality (VR) therapy to enable people with psychosis to practice skills in a computer-simulated world to reduce problems in real life. We have used similar treatment with people with worries (paranoia) about others and they have found it safe, acceptable and helpful. We have not used it for dealing with visions before.

The new treatment is delivered in two stages. First, a therapist will meet with you for about 4 sessions to try and understand with you how this experience developed and looks at different ways that you might try to manage the experience that would reduce any distress you experience or impact on your lifestyle. Your therapist will work through a series of topics on a computer that they think will help you. These topics cover a range of things, such as how the mind works, the types of experiences other people have and ways of coping that other people have found helpful. These can be done at your home or in a convenient clinic location.

Then we will offer you up to six sessions of virtual reality treatment. In these sessions you will work with your therapist and learn to put into practice the skills and ideas that were developed in the first few sessions. You will work in a room that allows us to show an image of your visual hallucination up on a projector or using a VR headset which may be an option if you prefer to do the treatment at home. It will help you practice dealing differently with this experience.

Who is funding the study?

The study is funded by the NHS National Institute of Health Research (NIHR) Research for Patient Benefit (RfPB)

Who has reviewed the study?

The study has been reviewed and given favourable opinion by the NHS East Midlands -Leicester South Research Ethics Committee Research Ethics Committee (Reference: (25/EM/0077)).

**Who is the Sponsor of the study?**

The study is sponsored by Cumbria, Northumberland, Tyne & Wear NHS Foundation Trust

**Who is managing the study?**

The Derby Clinical Trials Support Unit are managing the study.

**Do I have to take part?**

No. It is up to you to decide whether or not to take part. However, you might find it helpful to talk to others (e.g., a family member or a friend) about this. If you decide to take part, you will still be free to withdraw at any time, and you will not have to give a reason for withdrawing. If you decide not to take part, or if you withdraw from the study at a later date, the care you receive will not be affected in any way.

**What will happen to me if I take part?**

If you agree to take part, a researcher will arrange to meet you for your first study visit at either your home or a hospital clinic/team base at a mutually convenient time and then arrange to meet you for three further assessments. We think the assessments will last between 1 and 1.5 hours. They will involve you answering questions about your visions and other unusual sensory experiences (e.g., about how often you have them, what you think is happening), about your mood, about your quality of life (e.g., how satisfied you are with your financial situation, your work, and your relationships), and about your feelings about the therapy you received. In total, you will complete about 4-5 hours of assessments, spread over four sessions, if you decide to take part. Given how long the assessments take we would like you to be comfortable and you can take breaks and stop for a refreshment.

The first assessment will take place as soon as possible once you have agreed to take part in the study.

The second assessment (at your home or clinic/team base) will take place after you have completed the first part of the treatment but before you do the VR sessions, which we think will takes about 4-6 appointments with your therapist.

You will then meet for a third time about a month later (at 12 weeks, once again at your home or clinic/team base) after you have done the 4-6 VR sessions to complete another set of assessments. Then one month later (at 16 weeks) you will meet again for the final set of assessments (at your home or a convenient location).

We will also offer phone call check ins after each session to ask about wellbeing and to offer any support with in between session tasks (homework). So, we will actively monitor and support throughout the treatment sessions, will put in place strategies at the end of the session, will liaise with their broader care team and will check in with them in between sessions to check on progress, and wellbeing.

In total, therefore, you would be involved with the study for about 16 weeks.

We will ask you if it is ok to make an audio-visual recording of some of the therapy sessions. This is to help supervision and to ensure that you get the treatment we are trying to offer. At each session the therapist will ask you if it is ok to record the sessions and you can say no without giving a reason or stop at any time. It is your choice and entirely up to you. If agreeable, the session will be recorded on a trust encrypted laptop meaning it is secure. These audio-visual recordings may be used in supervision to help us deliver the best possible treatment and will be listened to by supervisor to check that the treatment is delivered as expected. The supervisor may ask one of the research team to listen to or view the recordings to check that the process is being done well. Anyone listening to or watching the session is bound by rules of confidentiality and no information about your own work and session content will be reported. It is a way for us to check we are doing things properly. We will only store the session~~s~~ recordings long enough to check the therapy, and they will be deleted once all participants have completed their study visits and assessments, which is the trial end date.

We might also get in touch to invite you to take part in a short interview after you had completed your therapy and before your final assessment sessions. We would like to ask about your views on participating in this study –particularly about your experiences of the assessment sessions and the new treatment that you had received. If you agree to be contacted for this you are not committed to doing it, and you can change your mind at any time, and we will explain more about it before you participate.

We will ask to record this follow up interview about your experience of the new treatment. The recording device is encrypted, to ensure that what you say is confidential and secure. Your views on the therapy are not shared with the therapist but are to only to help us to understand how helpful the treatment is and how we can improve it.

We will send audio recordings of the interviews (**but not any of the therapy sessions)** to a professional transcription company, used by and contracted to Cumbria, Northumberland,Tyne and Wear NHS Foundation Trust (CNTW). The transcriber will have signed a confidentiality agreement so must follow our rules about keeping your information safe. The audio file of the interview will be deleted after the transcription. The typed version of your interview will be stored securely and fully anonymised to remove any identifying information, other than a unique participant identification number and the use of your pseudonym (false name). The typed version of the interview will be kept for a minimum of five years.

We will also let your GP know you are taking part in this study, but this is for their information only

**How will my care be affected by taking part?**

In broad terms, the care you receive will not be affected by whether or not you decide to take part in this study. Your care team will decide with you what the most appropriate treatment is and offer you it, whether you participate or not. One difference is that the therapists will be able to receive supervision on the care offered to participants in the study, from the researchers.

With your permission, we will share a brief summary of their assessments with you and your clinical team, which may help your team in providing you with the appropriate support.

**Expenses and payments**

To thank you for taking part in this research and to compensate you for the time you will spend taking part in the study, you will be given £20 at each assessment (but not each therapy session). If you have to travel to meet with a researcher, your travel expenses will be reimbursed. This will be for both assessment and therapy sessions we can cover travel expenses.

**Are there any risks in taking part?**

There is a potential risk is that you may find it distressing to answer the researchers’ questions during your appointments with them. It is absolutely fine to take a break or stop the session if this occurs.

The treatment itself could cause some anxiety as you will be discussing your experiences of seeing things, and then the VR will provide a representation of the visual hallucination. You may find this upsetting. Most people do not find VR upsetting, but some can feel dizzy, or a bit tired afterwards. We will have a therapist with you at all times. You can tell us to stop showing the image of the vision, and we can spend time before and after each session helping you feel more at ease. You can have family or friends join you for the sessions as well.

**What are the possible benefits of taking part?**

The main benefit for you is the knowledge that you are taking part in research that is likely to help improve the care that is provided to people with visual hallucinations

**Will my taking part in the study be kept confidential?**

All of the information you provide in the study will be confidential. However, if you disclose information which indicates that you intend to harm yourself or others, confidentiality will have to be broken and we will have to inform the relevant authority (e.g., your care team). If you tell us about any criminal activity, we also will have to share this with the care team.

**How will we use information about you?**

We will need to use information from you and from your medical records for this research project.

This information will include your:

- Name
- Date of Birth
- Initials
- NHS record number
- Contact details
- Email address

People will use this information to do the research or to check your records to make sure that the research is being done properly.

People who do not need to know who you are will not be able to see your name or contact details. Your data will have a code number instead.  We will keep all information about you safe and secure.

Once we have finished the study, we will keep some of the data so we can check the results. We will write our reports in a way that no-one can work out that you took part in the study.

**What are your choices about how your information is used?**

- You can stop being part of the study at any time, without giving a reason, but we will keep information about you that we already have. .
- We need to manage your records in specific ways for the research to be reliable. This means that we won’t be able to let you see or change the data we hold about you.
- If you agree to take part in this study, you will have the option to take part in future research using your data saved from this study.

**Where can you find out more about how your information is used?**

You can find out more about how we use your information

- at [www.hra.nhs.uk/information-about-patients/](https://www.hra.nhs.uk/information-about-patients/)
- by asking one of the research team
- by sending an email to the study Chief Investigator: Dr Robert Dudley [rob.dudley@cntw.nhs.uk](mailto:rob.dudley@cntw.nhs.uk) or to the trial sponsor which is [CNTWSponsorManagement@cntw.nhs.uk](mailto:CNTWSponsorManagement@cntw.nhs.uk)
- By sending an email to the trust’s Data Protection Officer on [DPO@cntw.nhs.uk](mailto:DPO@cntw.nhs.uk)
- by ringing us on 0191 223 2020 and asking to speak with Dr Robert Dudley, the Chief Investigator for the study.

Cumbria, Northumberland, Tyne, & Wear (CNTW) NHS Foundation Trust is the sponsor for this study based in the United Kingdom and are working with the Open University and the Derby Clinical Trials Support Unit. We will be using information from you and your health records to undertake this study and CNTW will act as the data controller for this study. This means that we are responsible for looking after your information and using it properly. CNTW will keep identifiable information about you for five years after the study has finished.

Your rights to access, change or move your information are limited, as we need to manage your information in specific ways in order for the research to be reliable and accurate. If you withdraw from the study, we will keep the information about you that we have already obtained. To safeguard your rights, we will use the minimum personally identifiable information possible. You can find out more about how we use your information at <https://www.hra.nhs.uk/information-about-patients/>.

Researchers from CNTW NHS Foundation Trust will collect information from you and your medical records for this research study in accordance with our instructions. CNTW will keep your name and contact details confidential and will not pass this information to anyone outside of the Trust. CNTW will use this information as needed, to contact you about the research study, and make sure that relevant information about the study is recorded for your care, and to oversee the quality of the study. The people who analyse the information at CNTW will not be able to identify you and will not be able to find out your name or contact details.

All the data we collect will be stored on a password protected computer accessible only to the research team. Hard copies of consent forms and questionnaires that you provide will be kept in a locked cabinet at CNTW. Any audio recordings we make will be stored on an encrypted voice recorder before being transferred to a secure server at CNTW. Any study data you provide will be stored under an anonymous code and separately from personal data, to minimise the chance of anybody being able to identify you from your data. CNTW will keep identifiable information about you from this study for 5 years after the study has finished.

The insurance cover for the management, conduct and design of this study is provided under the NHS indemnity scheme.

**What will happen if I don’t want to carry on with the study?**

You are free to withdraw from the study at any time for any reason without affecting your future care or legal rights. If you chose to do so, we will use the information gathered up to the period of your withdrawal.

Your rights to access, change or move your information are limited, as we need to manage your information in specific ways in order for the research to be reliable and accurate. If your decision to withdraw is linked to the treatment being offered, we would ask that we can use this information, as this is important information about whether the treatment is acceptable to participants.

If you become very unwell during the study, you will be withdrawn from the study (i.e. you would stop taking part), and the Investigator will arrange for follow-up visits or telephone calls until you feel well again, and we would keep the information you had provided up to that point.

**What if there is a problem?**

If you are unhappy, or if there is a problem, please let us know by contacting Dr Robert Dudley, the Chief Investigator on 0191 232 2020 or (rob.dudley@cntw.nhs.uk) and we will try to help. If you remain unhappy or have a complaint which you feel you cannot come to us with, then you should contact Complaints and Patient Advice and Liaison Service Manager, on 0191 245 6679 or via complaints@cntw.nhs.uk. When contacting the Complaints Service, please provide details of the name or description of the study (so that it can be identified), the researcher involved, and the details of the complaint you wish to make. ￼

**What will happen to the results of the study?**

The results of the study will be submitted for publication in relevant scientific journals and may be presented at relevant conferences

At the end of the study, when all the data has been analysed, you will have the option to receive a lay / plain English summary of the results and outcomes.

No publication that uses data from this study will include your personal details or information that can identify you in any way.

**Further information and contact details**

If you have any further questions or things that you would like to see clarified before you decide if you want to take part or not, please free to contact our research team.

Dr. Robert Dudley,

Gateshead Early Intervention in Psychosis service

Grassbanks Health Centre,

Leam Lane,

Gateshead

Tyne & Wear

NE10 8DX

0191 232 2020

rob.dudley@cntw.nhs.uk

Consent form for Trial

**INFORMED CONSENT FORM FOR PARTICIPANTS IN VRV**

| **Version: 1.0** | **Date: 13/01/2025** |
| --- | --- |

**Study Title:** Virtual Reality for Visions (VRV): A proof-of-concept study examining the development of a new treatment for distressing visual hallucinations in people with psychosis

**Researcher:** Dr Robert Dudley

**Participant identification number for this study:** …………

|  | Please sign each box with your initials |
| --- | --- |
| I confirm that I have read and have understood the participant information sheet (version 1.0, dated 13/01/2025) for the above study. I have had the opportunity to consider the information, ask questions, and have had these answered satisfactorily by the researcher. |  |
| I understand that my participation is voluntary and that I am free to withdraw at any time without giving any reason and without my care or my rights being affected. |  |
| I agree to my General Practitioner being informed of my participation in the study. |  |
| I understand that information collected about me during the study may be looked at by the HRA/REC, by persons from the Trust and by individuals from Derby Clinical Trials Support Unit where it is relevant to my taking part in this study. I give permission for these individuals to have access to my records. |  |
| I agree for a brief summary of the assessments to be shared with my clinical team |  |
| I understand that the information collected about me will be used to support other research in the future, and may be shared anonymously with other researchers |  |
| I understand that the audio recordings of some interviews (but not therapy sessions) may be sent to a professional transcription company. We will anonymise these tapes where possible. However, there may be some identifying information discussed during the interview which may remain on the tape. The transcriber will sign a confidentially agreement so must follow our rules about keeping your information safe. |  |
| I agree to take part in the above study. |  |
| OPTIONAL:  I agree to be asked for the audio or visual recordings of each of my treatment sessions and can say no without giving a reason or stop at any time. I also understand how these recordings will be used and stored |  |

| Name of Participant | Date | Signature |
| --- | --- | --- |
| ———————————————— | ———— | ————————————— |
| Name of person taking consent | Date | Signature |
|  |  |  |
| ———————————————— | ——— | ————————————— |

**The contact details of lead researcher (Principal Investigator) are:**

Dr Rob Dudley, EIP Service (Gateshead) Gateshead Early Intervention in Psychosis service Grassbanks Health Centre, Leam Lane, Gateshead Tyne & Wear, NE10 8DX. Telephone:  0191 223 2020: [rob.dudley@cntw.nhs.uk](mailto:rob.dudley@cntw.nhs.uk)

***Three copies are collected for each consent process: one copy for site file, one copy for participant medical records, and one copy for the participant.***
