# Supplementary material for: Virtual reality for visions (VRV): a proof-of-concept study examining the development of a new treatment for distressing visual hallucinations in people with psychosis
Source: BMJ Open. 2026 Jan 12;16(1):e107535. doi: 10.1136/bmjopen-2025-107535 (PMC12815111; doi:10.1136/bmjopen-2025-107535)
Supplement: online supplemental file 2 [file bmjopen-16-1-s002.docx]

**Describing people’s visions**

Please use this flexibly and in conjunction with the manual that accompanies this. Please try and capture as much information as you can.

Agenda for the meeting

Introductions

*Introduce yourself, and your role in the study and confidential.*

Purpose of the session

*The aim of the session is to be able to identify a key visual experience to work on in treatment and to be able to describe it well enough that it can be developed by the programmers for a VR representation.*

Giving you control

*You are able to control the session, and what it is you talk about.*

Finding the right language (vision, visual hallucination, visual experience..)

*What does the person prefer to call them?*

Issues in assessment

*Normalise the experiences*

*Deal with the distress.*

Identifying what to work on (recent, distressing, common)

*Choosing the best example.*

Where do you tend to see the vision? What would be the most helpful for the treatment?

Story board the vision

**Scene 1: Where do you see the vision?***, (please describe if it is in a high street, bedroom, kitchen, garden etc, where they tend to see the vision most often.)*

*Please get a general description of where the person sees the vision*

(for example if a café, is it a small or large space, what is in there ( a counter, staff, customers, coffee machines, cakes and pastries, pictures on the wall, colours of the wall), what time of day is it, is it light or dark, and what sounds are noticeable (coffee machines, people chatting, cutlery sounds, door opening and closing).

**Where and when does the vision occur?**

- **Location** (when you see the vision is it directly in front of them, beside them, in a specific part of the room):
- **Time of day** (e.g. night, early morning, so is it dark, bright light etc):
- **Lighting** (e.g. bright sunlight, dim, artificial light):

**Scene 2: what is that you see?**

Here we need to know what it is that they see. We need the detail to be able to develop a description of the vision.

**Content: Can you tell me a bit about what you see?**

*Please get a general description of the vision:*

(consider size, shape, location, colour, if a person details like hair colour, teeth, smiling or frowning, clothing, capture as much detail as possible).

**Familiarity**: If it is a person or an animal or object do you recognise it?

- ☐ Known person/object
- ☐ Unknown
- ☐ Imagined/fictional (like a character from a story or a film?)

**Coherence: Can you tell me a bit more about the vision?**

*(Please tick all that apply and elaborate below)*

Realism: Does it look like a normal person/object and you see it in three dimensions or is it more like a photo or a painting in two dimensions? Is it like a real object or a cartoon?

- ☐ 2D
- ☐ 3D
- Does the vision blend in with the real world or appear separately? So it is standing out, and not connected to or fitting in with the surroundings? So would it be sat at a chair or hovering about the floor?
  - ☐ Blended with surroundings
  - ☐ Not blended, rather it is distinct and separate

**Movement**. Does the vision move or does it remain still?

- ☐ Still
- ☐ Moving (please describe how it moves, such as appearing where-ever the person looks?)

**Sound**: When you see it do you hear anything at the same time? Does the vision have a sound or does it speak to you, or is it a silent image? Are there other sounds that are important, laughter, screaming, a rustle of the wind, or a slamming door that happen at the same time as you see the vision?

- ☐ No sound
- ☐ Sound present (what do you hear and where it’s coming from):

**Tactile**: Does the vision interact with you, can you feel it stroke or touch you, or push or grab you? Does it feel hot or cold?

- ☐ No tactile
- ☐ Tactile experiences present

**Olfactory**: Does the vision have a smell or do you smell things when you see the vision?

- ☐ No olfactory
- ☐ Olfactory experiences present

**Quality**

- Mostly visions look realistic and life like but is there anything about it that does not seem quite right to you? It looks a bit different to what you would expect (face missing some features, the movement is not right, the clothes it wears appears different).

☐ Uncanny valley features

**Contextual Information**

**Frequency/duration** (e.g. how often and how long does it last?):

**Temporal and Dynamic Features**

**Are there any changes or patterns in how the vision appears over time?**

*(e.g. does it change from one thing to another or, fade, intensify, move closer?)_

**Scene 3: what makes it distressing?**

To make the treatment effective though we need to know what it is about the vision that is the most upsetting. Here imagine we are zooming in on the worst aspect of seeing the vision. Ask the person as if it was a hotspot memory about what it is that they pay most attention to. It could be some aspect of the uncanny valley features mentioned above (the face has no features) or it could be the way it moves (suddenly towards you), or a change in the expression on the face, or a way it glances at you.

**When it is at the worst, what is that you notice about the vision?**

*Please get a specific description of the vision:*

(Consider here if it is a change in the vision, does it say something, does it approach the person, have a look in its eyes?)

**Preliminary formulation of the vision**

Try and fill in the detail about the vision in terms of the CHAP formulation model

**Additional Notes for VR Design**

**Any additional details that may help develop the VR model?**

Is there anything about the vision that is very distinctive or really important to be aware of when developing a VR representation?

**What is most important to capture about the experience?**

*(Textures, temperature, smell, lighting source, environmental context, etc.)*
