# Supplementary material for: Virtual reality for visions (VRV): a proof-of-concept study examining the development of a new treatment for distressing visual hallucinations in people with psychosis
Source: BMJ Open. 2026 Jan 12;16(1):e107535. doi: 10.1136/bmjopen-2025-107535 (PMC12815111; doi:10.1136/bmjopen-2025-107535)
Supplement: Supplementary file 3 [file bmjopen-2025-107535.reviewer_comments.pdf]

PEER REVIEW HISTORY

BMJ Open publishes all reviews undertaken for accepted manuscripts. Reviewers are asked to complete a checklist review form and are provided with free text boxes to elaborate on their assessment. These free text comments are reproduced below.

ARTICLE DETAILS

Title (Provisional)

Virtual Reality for Visions (VRV): A proof-of-concept study examining the development of a new treatment for distressing visual hallucinations in people with psychosis.

Authors

Dudley, Robert; Sargeant, Samuel; Gibbs, Christopher; Prentice, Louise; McCartney, laura; Aynsworth, Charlotte; Maskey, Morag; Skeggs, Andrew

---

VERSION 1 - REVIEW

**Reviewer**	**1**
**Name**	**Rus Calafell, Mar**
**Affiliation**	**Ruhr-Universitat Bochum, Mental Health Research and Treatment Centre, Faculty of Psychology**
**Date**	**30-Oct-2025**
**COI**	**None**

Thank you for inviting me to review the paper entitled " Virtual Reality for Visions (VRV): A proof-of-concept study examining the development of a new treatment for distressing visual hallucinations in people with psychosis". This manuscript presents the study protocol of a single-arm (non-controlled) protocol evaluating a novel virtual-reality (VR) intervention targeting distressing visual hallucinations in people with psychosis. The topic is innovative and potentially high-impact; if feasible and acceptable, it could provide unprecedented evidence for applying VR to these symptoms. Several clarifications would strengthen the protocol's transparency, reproducibility, and regulatory alignment.

I have some major comments, especially in regard to the intervention:

1. Intended use / regulatory scope of the XR platform (psychosis & visual hallucinations): The manuscript (p. 17) states that the XR Therapeutics technology is indicated for psychosis-specifically for visual hallucinations. However, the XR Therapeutics website does not (at least prominently) list psychosis among its target conditions. This inconsistency raises the question of whether, in this trial, the device is being used within its intended purpose or outside its stated scope. Please clarify:

a) What is the official intended use of the device for this study? Is psychosis/visual hallucinations explicitly covered?
b) If within scope, provide a verifiable source (e.g., webpage URL with access date, indication statement) and/or quote the relevant wording.
c) If outside the intended purpose, please describe how the investigation complies with applicable medical-device regulations (e.g., provisions governing clinical investigations conducted for purposes other than conformity assessment) within the main text of the manuscript.

2. Nature and personalization of VR stimuli: the manuscript should specify the stimulus set used in the VR exposure/interaction phase:
   a) Categories (e.g., human figures, objects, animals, shadow-like forms, lights/patterns), modalities (visual only vs. audio-visual), and behavioural dynamics (static vs. animated; scripted vs. interactive).
   b) Personalization: To what extent are stimuli tailored to the individual's idiosyncratic hallucinations? Can the system reproduce specific features (e.g., size, colour, motion, distance, luminescence, level of detail)?
   c) If personalized, how are assets created or configured (e.g., therapist-guided elicitation and rapid prototyping within session; pre-session asset creation; use of libraries vs. custom models; who signs off on fidelity)?

3. Hardware setup and immersion (minor comment): Please state whether the VR suite is immersive Cave Automatic Virtual Environment (CAVE), or another set-up (even 2D?)

VERSION 1 - AUTHOR RESPONSE

Reviewer: 1
Prof. Mar Rus Calafell, Ruhr-Universitat Bochum, King's College London
Comments to the Author:
Thank you for inviting me to review the paper entitled " Virtual Reality for Visions (VRV): A proof-of-concept study examining the development of a new treatment for distressing visual hallucinations in people with psychosis".
This manuscript presents the study protocol of a single-arm (non-controlled) protocol evaluating a novel virtual-reality (VR) intervention targeting distressing visual hallucinations in people with psychosis. The topic is innovative and potentially high-impact; if feasible and acceptable, it could provide unprecedented evidence for applying VR to these symptoms. Several clarifications would strengthen the protocol's transparency, reproducibility, and regulatory alignment.

I have some major comments, especially in regard to the intervention:
1. Intended use / regulatory scope of the XR platform (psychosis & visual hallucinations): The manuscript (p. 17) states that the XR Therapeutics technology is indicated for psychosis-specifically for visual hallucinations. However, the XR Therapeutics website does not (at least prominently) list psychosis among its target conditions. This inconsistency raises the question of whether, in this trial, the device is being used within its intended purpose or outside its stated scope. Please clarify:
a) What is the official intended use of the device for this study? Is psychosis/visual hallucinations explicitly covered?

We appreciate the careful consideration of this issue. We have included the Declaration of conformity that was submitted to MHRA for device registration. As you can see this covers all mental health conditions and talking therapies. We worked closely with the company and a medical device consultant who confirmed to us that this

would include people with mental health issues and fears including for adults (16+) attending psychosis services who have distressing visual hallucinations. We have removed this final statement from the text and have instead used the wording in the device registration as this then provides clarity on the approved status of the device.

b) If within scope, provide a verifiable source (e.g., webpage URL with access date, indication statement) and/or quote the relevant wording.

Please see above, we have provided the MHRA declaration of conformity.

c) If outside the intended purpose, please describe how the investigation complies with applicable medical-device regulations (e.g., provisions governing clinical investigations conducted for purposes other than conformity assessment) within the main text of the manuscript.

We understand from the declaration of conformity that all mental health conditions are covered.

2. Nature and personalization of VR stimuli: the manuscript should specify the stimulus set used in the VR exposure/interaction phase:
a) Categories (e.g., human figures, objects, animals, shadow-like forms, lights/patterns), modalities (visual only vs. audio-visual), and behavioural dynamics (static vs. animated; scripted vs. interactive).

In the exposure/VR interaction phase we create representations of the visual experience reported. It is expected this will often be figures, shadow figures, etc. We are able to make the vision move around the scenario that it is experienced in. We are able to program it to make relevant noises, utter speech but this is scripted and not interactive.

This has been added to page 14 para 3.

b) Personalization: To what extent are stimuli tailored to the individual's idiosyncratic hallucinations? Can the system reproduce specific features (e.g., size, colour, motion, distance, luminescence, level of detail)?

On page 14 para 3

"The initial session elicits a detailed description of the VH which will be shared with the VR programmers to allow a person specific representation of their VH to be developed "

So yes, the scenarios are personalised. We aim to reproduce something approximating the person's unique visual experience. So, as described below we capture information about size, shape, motion, distinguishing features etc. to create a bespoke representation.

c) If personalized, how are assets created or configured (e.g., therapist-guided elicitation and rapid prototyping within session; pre-session asset creation; use of libraries vs. custom models; who signs off on fidelity)?

At an assessment session we story board the VR scenario. We ask where the person most often sees the vision and capture relevant detail about this (a bedroom, living room, kitchen, street, etc), and detail relevant information about it (layout of room, noises, lighting etc). This is the setting in which people will enter the VR. Then we ask about key information about the vision (size, colour, transparency, movement, sounds, if the vision is of a person then we ask about hair colour, facial features, etc). At this stage we may ask the person to describe or draw the image, or we can use images generated from google searches to help check on key characteristics (ie if a shadow figure, how dark is the shadow etc). Then we ask if there is a specific feature of the vision that is most relevant to capture. It can be a type of movement, or a way it interacts with the person (such as stopping and staring, moving to or away from the person). This assessment process was developed by our PPI group, and captured on an assessment tool that was reviewed by the ethics committee, and a copy of which is provided now as a supplementary material.

This VR representation is created by the VR company and then in the first session with the participant we ask about the perceived credibility of the vision to check it is seen as a viable/realistic representation of the experience. This is one of our feasibility aims to determine if this can be used to develop a relevant personalised experience. The scene in which they see the vision will be built/adapted from a library of rooms/settings. We enable people to view the room from different angles/viewpoints and for the lighting, and background noise to be adjusted to increase the realism of the environment. We typically allow for the image to made more or less transparent, and smaller or larger in size, and this is controlled by the participant. So, the image they start with maybe less like their vision as it is smaller, or more see through, but the person is able to increase the realisticness of it (more opaque, full size etc), and we ask about the credibility of the images that are used.

3. Hardware setup and immersion (minor comment): Please state whether the VR suite is immersive Cave Automatic Virtual Environment (CAVE), or another set-up (even 2D?)

As we state on page 16 last para we have the option to use VR via headsets to create the immersive 3D setting, or via projection on to a screen or large TV screen presentation (so it is 3D environments presented on a large 2D screen or TV). The aim is to explore the preferred use of this approach. Large screens are helpful for therapist and participants to work alongside each other, and are more practical than a CAVE system but does still require access to the equipment, whereas the headsets are more easily transported and used in people's homes or clinics. For this proof of concept study we are mainly using the large screen where we can so that we are able to learn about the best use of the VR.

Thank you for your careful consideration of the protocol and we hope the above addresses the important issues raised,

Reviewer: 1
If you have selected 'Yes' above, please provide details of any competing interests.: Not applicable.
